# Astragaloside IV alleviates senescence of vascular smooth muscle cells through activating Parkin-mediated mitophagy

**DOI:** 10.1007/s13577-022-00758-6

**Published:** 2022-08-04

**Authors:** Huijun Li, Jialin Xu, Yanan Zhang, Lei Hong, Zhijian He, Zhiheng Zeng, Li Zhang

**Affiliations:** 1 Department of Cardiology, The First Affiliated Hospital of Guangdong Pharmaceutical University, No. 19, Nonglinxia Road, Yuexiu District, Guangzhou, 510080 Guangdong China; 2 Key Laboratory of Animal Virology of Ministry of Agriculture, Center for Veterinary Sciences, Zhejiang University, Hangzhou, China; 3 Department of Cardiology, Long Gang Central Hospital of Shenzhen, Shenzhen, 518116 Guangdong China

**Keywords:** Astragaloside IV, Senescence, Vascular smooth muscle cells, Parkin, Mitophagy

## Abstract

Astragaloside IV (AS-IV), as one of the main active components of *Astragalus membranaceus*, has been reported to have cardiovascular protective effects. However, the role and molecular mechanism of AS-IV in vascular senescence have not been clearly stated. The in vitro aging model was constructed using bleomycin (BLM) in vascular smooth muscle cells (VSMCs). Cell senescence were assessed through Western blotting analysis of aging markers, flow cytometry, and the β-galactosidase (SA-β-Gal) kit. Mitophagy was determined through transmission electron microscopy, TMRM staining, and Western blotting analysis of p62. A model of aging blood vessels was induced by d-gal. The vascular wall thickness of mice was also evaluated by H&E staining. Our data proved that AS-IV plays an anti-senescent role in vitro and in vivo. Results showed that AS-IV effectively improved mitochondrial injury, raised MMP, and mediated mitophagy in BLM-induced senescent VSMCs and d-gal induced aging mice. Parkin expression strengthened AS-IV’s anti-senescent function. In conclusions, AS-IV attenuated BLM-induced VSMC senescence via Parkin to regulate mitophagy. Therefore, AS-IV-mediated Parkin might be a latent therapeutic agent and target for VSMC senescence.

**Supplementary Information:**

The online version contains supplementary material available at 10.1007/s13577-022-00758-6.

## Introduction

Senescence is a prevalent phenomenon in nature and is a necessary procedure for organisms [1]. With the aging of the global population, the incidence of cerebrovascular and cardiovascular diseases (CVDs) such as atherosclerosis, myocardial infarction, hypertension, heart failure, and stroke, has dramatically increased [2, 3]. Based on 2015 data from the United States, the average incidence of first-episode cardiovascular events in people aged 80 years was 74 [4]. Study confirmed that vascular smooth muscle cell (VSMC) senescence is the main pathological mechanism of CVDs [5]. Senescent VSMCs are characterized by growth arrest, cytoskeleton stiffness, and increased secretion of inflammatory factors [6]. Study proved that senescent VSMCs can be detected in human atherosclerotic plaques, but they are almost undetectable in normal vascular tissues [7]. By secreting multiple cytokines and growth factors, senescent VSMCs can change the local microenvironment of tissues, induce inflammatory responses and vascular sclerosis, and aggravate the process of related diseases [8]. However, the occurrence and development mechanism of VSMC senescence still lacks in-depth study.

Autophagy is a key way for cells to clear senescent or damaged organelles or proteins, which is essential for maintaining homeostasis [9]. Mitophagy refers to the process by which cells selectively clear mitochondria through the mechanism of autophagy [10]. In the early stage of disease, mitophagy can maintain cell homeostasis in a compensatory way, but long-term mitophagy results in cell decompensation and apoptosis [11]. Abnormal mitophagy is closely associated with cellular senescence [12]. Under normal conditions, mitophagy can clean up mitochondria that have lost their function, thereby protecting cells and preventing senescence [13]. In the aging state, the function of mitophagy declines, causing the gradual accumulation of damaged mitochondria, which further aggravates the senescence process [14]. Moderate autophagy is crucial to ameliorate mitochondrial dysfunction and delay the senescence of VSMCs [15]. Therefore, mitophagy may be a vital target for the therapy of VSMC senescence. Further exploration of therapeutic drugs that can affect mitophagy is crucial to VSMC senescence.

Astragalus membranaceus (AM), as a traditional Chinese medicine, has the functions of strengthening the exterior and reducing sweat, healing sores, reinforcing Qi and promoting diuresis [16]. AM also has multiple pharmacological activities such as antioxidation, immunomodulation, anti-inflammation and anti-ischemic brain injury [17, 18]. Astragaloside IV (AS-IV) is the main active saponin ingredient extracted from AM [19]. Pharmacological studies also verified that AS-IV has anti-inflammatory, anti-oxidation, anti-apoptosis, and other pharmacological effects [20]. Moreover, AS-IV has liver protection, anti-aging, anti-stress, anti-hypertension, and antibacterial properties [21, 22]. Study also found that AS-IV can increase the content of superoxide dismutase (SOD) in cardiomyocytes. However, it is still not completely clear whether AS-IV plays an anti-aging role by regulating mitophagy in VSMCs.

In the current study, we further uncovered the therapeutic effect of AS-IV on VSMC senescence, and preliminarily verified the influences of AS-IV on mitochondrial dysfunction and autophagy. Moreover, we also analyzed the possible regulatory mechanism of AS-IV in BLM-induced VSMCs. Thus, we confirmed the protective role and mechanism of AS-IV on VSMC senescence, suggesting that AS-IV might be a novel therapeutic drug for VSMC senescence.

## Materials and methods

### Cell culture

Human vascular smooth muscle cells (hVSMCs) were purchased from BeNA Culture Collection (BNCC332960). hVSMCs were cultured in F-12 K (ATCC, Catalog No. 30-2004) complete growth medium. To make the complete growth medium, the following components were added to the F-12 K base medium: 0.05 mg/ml ascorbic acid, 0.01 mg/ml insulin, 0.01 mg/ml transferrin, 10 ng/ml sodium selenite, 0.03 mg/ml Endothelial Cell Growth Supplement (ECGS), fetal bovine serum (Unovl Biotechnology, 107-FBS-500) to a final concentration of 10%, HEPES to a final concentration of 10 mM, and TES to a final concentration of 10 mM. Cells were cultured at 37 °C and 5% CO_2_.

### Animal and model of aging

Male BABL/C mice of SPF grade (6 weeks, weight: 18 ± 20 g) were provided by the Experimental Animal Center of Guangdong Pharmaceutical University. All mice were kept in the SPF animal laboratory at a constant temperature of 18–20 °C, a humidity of 50%–80%, sterile water, and standard feed. The animal experiments conformed the Guidelines of the International Committee on Laboratory Animals. The experimental animal study was approved by The First Affiliated Hospital of Guangdong Pharmaceutical University (approval NO. gyfygzr030).

To construct an in vivo model of vascular aging, d-galactose was used in this experiment. Fifty mice were randomly divided into 5 groups: the control group, aging model group (d-gal), AS-IV low concentration group (d-gal + LD-AS-LV), AS-IV medium concentration group (d-gal + MD-AS-LV), and AS-IV high concentration (d-gal + HD-AS-LV) group. Except for the control group, the other four groups received D-galactose daily for 8 weeks at a dose of 150 mg/kg/day. While receiving the d-galactose injection, the animals were treated with astragaloside IV (Sigma) by gavage. Specifically, two weeks after receiving the first d-galactose injection, the animals received daily intragastric administration of astragaloside IV at doses of 20, 40, and 80 mg/Kg for 2 weeks. At the end of the experiment, the animals were anesthetized with pentobarbital and subjected to systemic vascular perfusion. Subsequently, the animals in each group were dissected and the blood vessels were collected for subsequent experiments.

### Cell treatment

Parkin-overexpressed plasmids and Parkin shRNAs were purchased from HanBio Biotechnology (HanBio, Shanghai, China). In brief, BLM-induced VSMCs cells were transfected with these plasmids using Lipofectamine 3000 (Invitrogen) in accordance with the detailed specification. BLM-induced VSMCs were given different doses of AS-IV (10, 50, or 100 μM) and Mdivi-1 (1 μM). After 48 h of treatment with AS-IV, plasmids, or other drugs, the cells were collected for subsequent assays.

### Mitochondria isolation

Mitochondria and cytosolic fractions were isolated from cells and tissues with a commercially available kit (89,874, Thermo Fisher Scientific, Waltham, MA, USA) according to the manufacturer’s instructions.

### Western blot

The treated VSMCs and the aorta of mice were collected from each group, and the total proteins were extracted through the use of RIPA lysis buffer (Beyotime, China). After quantification, the total protein (20 μg) in each group was subjected to electrophoresis and transferred onto the prepared PVDF membranes (Millipore). Then the membranes were exposed to 5% skim milk for 1.5 h, incubated with rabbit anti- primary antibodies overnight at 4 °C, and goat anti- secondary antibodies (Abcam, ab6721, 1:10,000) for 2 h. The protein blots were observed using the chemiluminescent reagent (Millipore, Burlington, MA, USA). The primary antibodies included p16 (Beyotime, AF1069, 1:1000), p21 (ProteinTech, 10,355–1-AP, 1:3000), DcR2 (Boster, A05136, 1:1500), P62 (ProteinTech, 18,420–1-AP, 1:3000), TOMM20 (Boster, BM4366, 1:2000), Drp1 (Abcam, ab184247, 1:1000), and Parkin (Boster, PB9307, 1:1500). GAPDH expression was set as the internal reference.

### Transmission electron microscopy (TEM)

The collected samples were fixed using 2.5% glutaraldehyde at 4 °C, dehydrated with acetone, and embedded. The embedded tissues were sliced into 80 nm slices and placed in a copper mesh. After staining with lead citrate and uranium acetate, the ultrastructure of the tissue samples was observed using a JEM-100CX microscope (JEOL, Japan).

### TMRM staining and detection

The treated VSMCs were incubated at 37 °C for 45 min in a solution containing 2 mM CaCl_2_, 1.25 mM KH_2_PO_4_, 2 mM MgSO_4_, 3 mM KCl, 156 mM NaCl, and the TMRM probe (25 nM). The cells were subsequently observed using laser confocal microscopy. The positive cells were detected by flow cytometry (parameters: excitation wavelength 543 nm, generation wavelength 605 nm). The results were analyzed with FlowJo software (BD, USA).

### Flow cytometer

Cells were fixed with paraformaldehyde, and then SPiDER-βGal working solution was added, and the samples were incubated at 37 °C for 30 min. The cells were then digested with trypsin and resuspended in MEM medium and detected by flow cytometry (parameters: excitation wavelength 488 nm, generation wavelength 530 nm). The results were confirmed with FlowJo software (BD, USA).

### H&E staining

The aorta of mice was first subjected to a series of treatments, including fixation (4% paraformaldehyde), dehydration (gradient ethanol), and embedding in paraffin. Then the tissues were cut into continuous 4 μm slices. The slices were dewaxed with xylene I, xylene II, dehydrated with 95%, 90%, 80%, and 70% ethanol, and washed with distilled water. Then the slices were processed with Harris hematoxylin, 1% hydrochloric acid alcohol, 0.6% ammonia, and eosin. After dehydration (gradient ethanol) and transparency (xylene), the pathological structure was observed with a microscope.

### Immunofluorescent (IF) assay

Briefly, immunofluorescent staining of the treated VSMCs and aorta of mice in fixed paraffin sections was used to analyze the distribution of p16, p21, and Parkin proteins. Microwave ovens were used for antigen retrieval in a citrate solution for 20 min. Endogenous peroxidase activity was blocked using 3% hydrogen peroxide for 15 min and rinsing in PBS. The glass slides that had crawled cells were sealed with sealing solution (5% donkey serum, Solarbio Science and Technology Co., Ltd., Beijing, China) was dripped at room temperature for 30 min. Each slide had a sufficient amount of diluted primary antibodies (p16: Beyotime, AF1069, 1:100); p21 (ProteinTech, 10,355–1-AP, 1:3000); Parkin (Boster, PB9307, 1:300) dripped on it and then it was put into a wet box, and incubated at 4 °C overnight, goat anti-rabbit secondary antibodies (Abcam, ab150077, 1:500) or goat anti-rabbit secondary antibodies, (Proteintech, SA00013-2, 1:200) were added for 1 h at room temperature. Cell nuclei were stained with 4′,6-diamidino-2-phenylindole (DAPI; Sigma-Aldrich, CA, USA). Fluorescent images were captured using a fluorescent microscope system (Leica, Germany).

### Enzyme-linked immunosorbent assay (ELISA)

Blood samples from mice were collected, and centrifuged to obtain serum. The concentration of β-galactosidase was tested with the β-Gal ELISA kit (Meimian Biotechnology, MM-1118M2) in line with the manufacturers’ instructions.

### Statistical analysis

All data from three independent experiments were displayed as the mean ± SD. Data analysis was conducted using SPSS 20.0 software (SPSS, Inc.) with one-way ANOVA. *P* < 0.05 denoted that the statistical result was statistically significant.

## Results

### AS-IV ameliorated the senescence of BLM-induced VSMCs

**Fig. 1:**
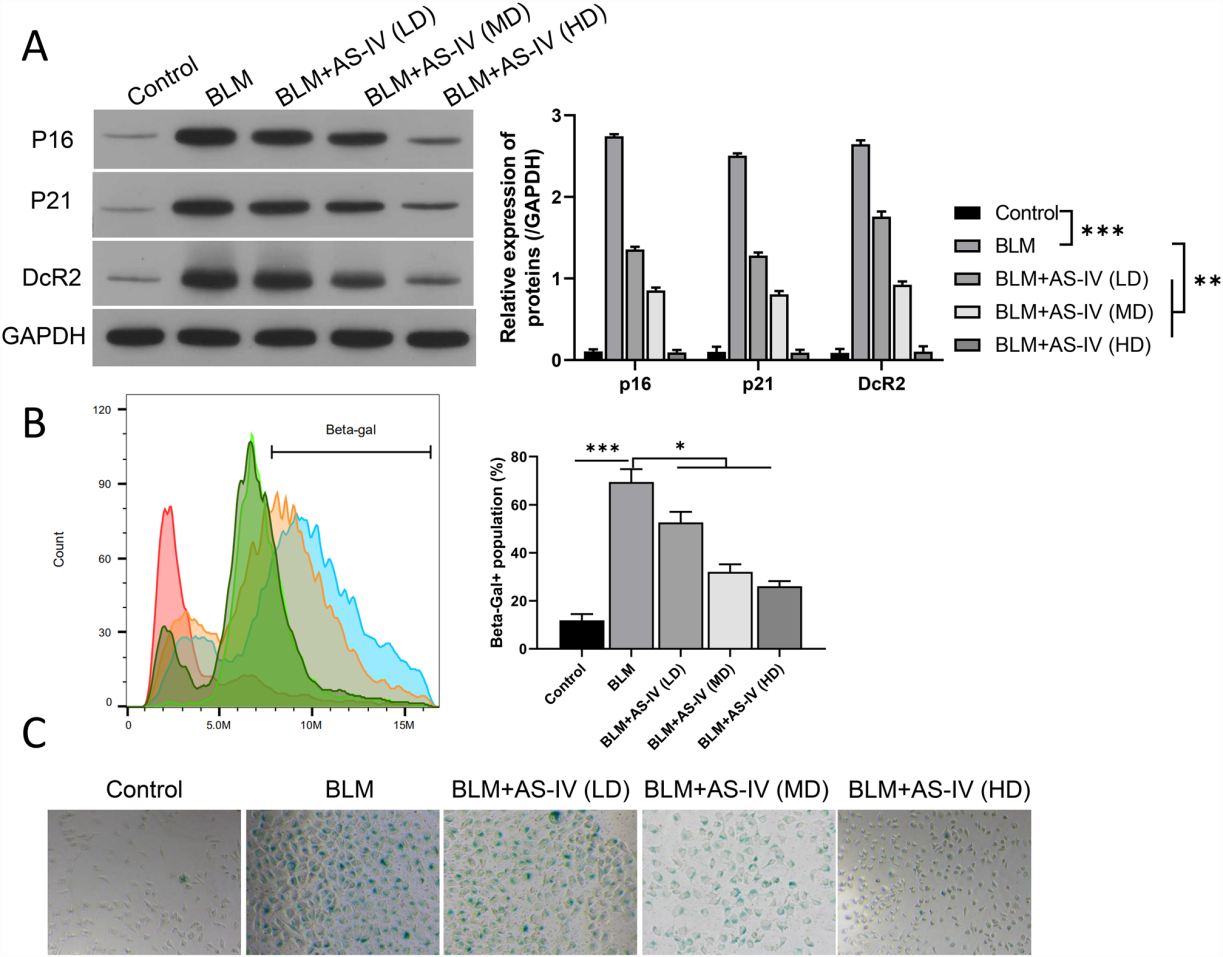
AS-IV ameliorated the senescence of BLM-induced VSMCs. BLM-treated VSMCs were given different doses of AS-IV. **A** Western blot exhibited the expression changes of p16, p21, and DcR2, and the protein levels were quantified on the gray scale. **B** The population ofβ-galactosidase was tested using flow cytometer, and quantitative analysis was conducted. **C** After processing with AS-IV, the SA-β-galactosidase kit was used to evaluate the senescence of BLM-induced VSMCs. Magnification, 200 × , scale bar = 50 μm. *L* low dose, *M* medium dose, *H* high dose. *P < 0.05, ***P < 0.001

To investigate the preventive effect of AS-IV on VSMC senescence, we first used BLM to induce the senescence of VSMCs, which were also given different doses of AS-IV. Western blotting data showed that expressions of p16, p21, and DcR2 were raised in the BLM group compared with the control group, while the upregulation of p16, p21, and DcR2 expressions was markedly weakened by the introduction of AS-IV in a concentration dependent manner **(**Fig. 1A). Flow Cytometer data showed that the percentage of β-galactosidase positive cells in VSMCs was elevated in the BLM-treated VSMCs, while treatment with AS-IV dramatically reduced the amount of β-galactosidase induced by BLM in a concentration dependent manner (Fig. 1B). Next, results of β-galactosidase staining showed that the senescence of VSMCs (cells in blue) was increased in the BLM group compared to the control group, while addition of AS-IV attenuated the increase of SA-β-galactosidase in BLM-induced VSMCs (Fig. 1C). In general, these results proved that BLM induced senescence-like VSMCs, and AS-IV has a prominent relieving role on VSMC senescence induced by BLM.

### AS-IV protected VSMCs from senescence by mediating mitochondrial quality and mitophagy

**Fig. 2:**
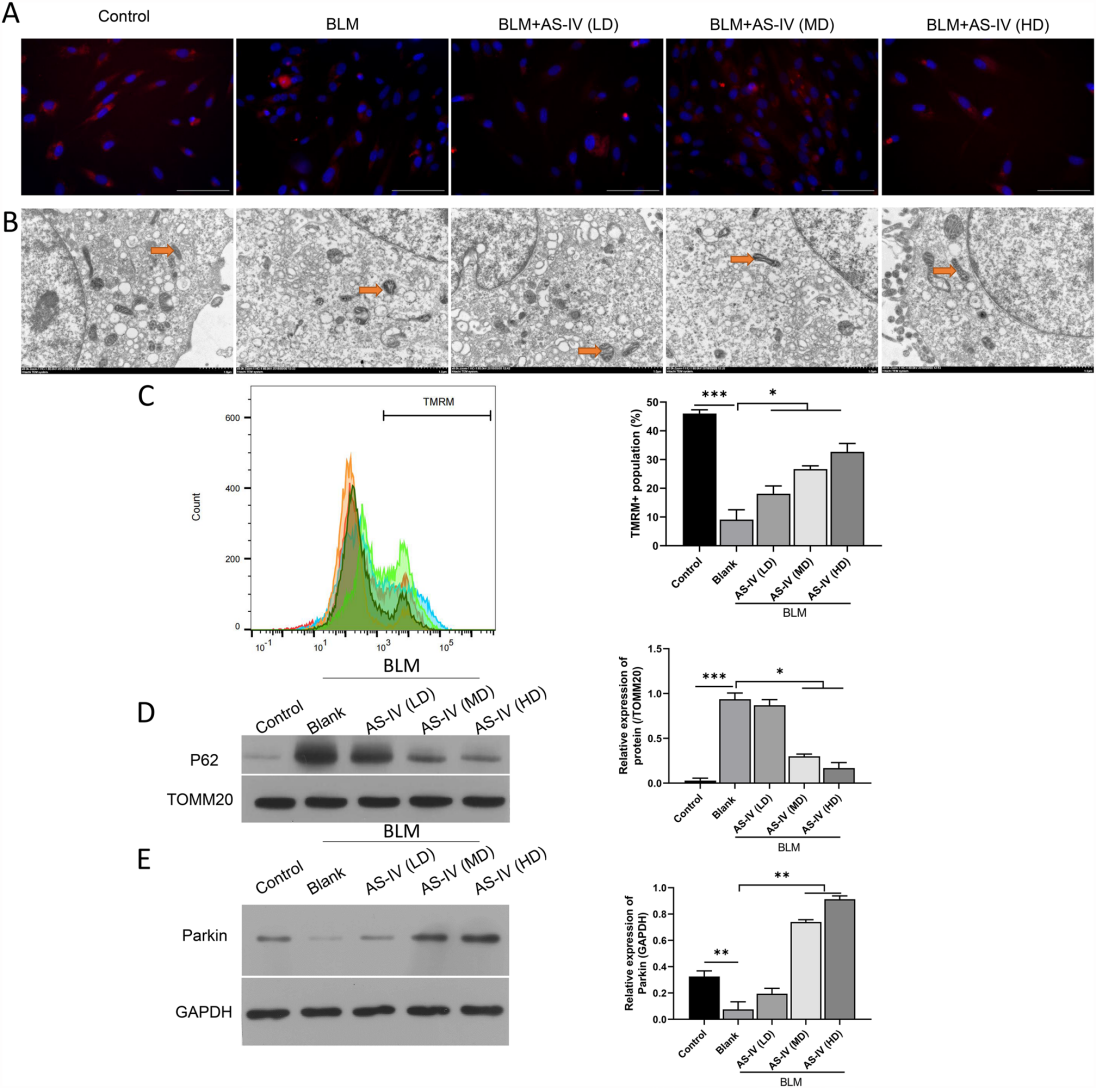
AS-IV improved mitophagy and attenuated mitochondrial membrane potential loss in BLM-induced VSMCs. **A** After administration of AS-IV, MMP was identified by TMRM staining in BLM-induced VSMCs. Magnification, 200 × , scale bar = 50 μm. **B** TEM was used to observe the influence of AS-IV on the mitochondrial structure of BLM-induced VSMCs. **C** Flow Cytometer was used to assess the impact of AS-IV on the TMRM + fluorescent intensity in BLM-stimulated VSMCs. **D** P62 expression was examined by Western blot in BLM-induced VSMCs, which were given AS-IV, and the P62/TOMM20 value was determined. **E** expression of Parkin was measured by Western blot in BLM-induced VSMCs. *LD* low dose, *MD* medium dose, *HD* high dose. *P < 0.05, **P < 0.05, ***P < 0.001

Subsequently, we further verified the impacts of AS-IV on mitochondrial injury and autophagy of BLM-stimulated VSMCs. TMRM-staining data first indicated that MMP was lost in the BLM group, while AS-IV could markedly aggrandize the MMP of BLM-induced VSMCs (Fig. 2A). Then TEM results showed that AS-IV promoted mitophagy (yellow arrow) in BLM-induced senescent VSMCs. Specifically, there was swelling in mitochondrial compartment, dissolution, shedding, and decrease of mitochondrial cristae in BLM-induced VSMCs. However, the results of TEM showed that the morphology of mitochondria in BLM-induced cells gradually recovered with the treatment of AS-IV **(**Fig. 2B, yellow arrow). In order to quantitatively analyze the mitochondrial membrane potential of the cells in each group, the cells were treated with the TMRM probe, and then the potential was detected by flow cytometry. As shown in Fig. 2C, the loss of membrane potential was significantly increased after BLM treatment. As the concentration of AS-IV increased, the loss of MMP was mitigated (Fig. 2C). To further confirm the level of mitophagy, Western blot was used to detect the expression level of P62, the substrate of mitophagy. As shown in Fig. 2D, the expression level of P62 was significantly upregulated in VSMCs treated with BLM, while AS-IV effectively reduced the P62 expression level. While, BLM suppressed expression of Parkin in VSMCs and AS-IV treatment recovered the expression of Parkin (Fig. 2E). To sum up, the data showed that AS-IV can notably increase MMP and mediate autophagy in BLM-induced VSMCs.

### Mitophagy inhibitor, Mdivi-1, effectively affected the anti-senescent function of AS-IV.

**Fig. 3:**
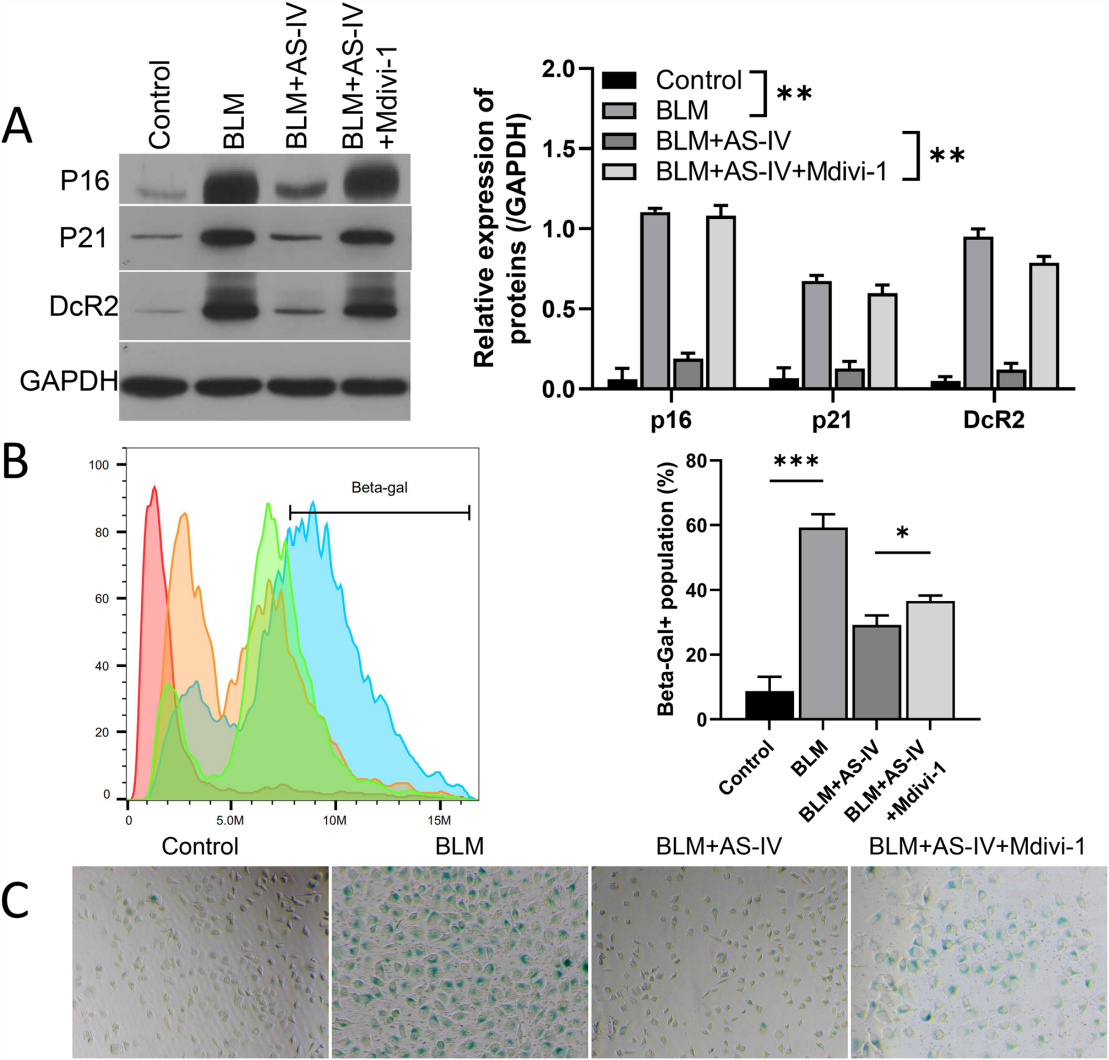
Mitophagy mediators regulated the functions of AS-IV on BLM-induced VSMC senescence. BLM-treated VSMCs were given AS-IV, and then exposed to the autophagy inducer (Torin 1) or mitophagy inhibitor (Mdivi-1). **A** Western blot was used to assess the protein levels of P62, p21, and DCR2, and quantitative analysis of the protein was also determined. **B** Flow Cytometer was used to analyze the population ofβ-galactosidase in each group. **C** Cellular senescence was evaluated using a SA-β-galactosidase kit in each group. Magnification, 200 × , scale bar = 50 μm. *P < 0.05, **P < 0.01, ***P < 0.001

Based on the roles of AS-IV in the maintenance of mitochondrial fusion and division balance, and the induction of autophagy in BLM-induced VSMC, we further investigated whether autophagy mediators exert a key effect on the senescence of VSMCs. Thus, after treatment with AS-IV, we used the mitophagy inhibitor (Mdivi-1) to treat BLM-treated VSMCs. As shown in Fig. 3A, Mdivi-1 up-regulated p16, p21, and DcR2 in BLM-induced VSMCs. (Fig. 3A). Flow cytometry showed that the loss of mitochondrial membrane potential of VSMCs induced by BLM was higher after Mdivi-1 treatment than in the AS-IV group (Fig. 3B). The results of β-galactosidase staining showed that the aging characteristics of VSMCs induced by BLM were alleviated after AS-IV treatment. However, AS-IV showed up-regulation of senescent characteristics after Mdivi-1 treatment (Fig. 3C). In short, the mitophagy mediator played a key regulatory role in BLM-induced senescent-like VSMCs.

### Mitophagy inhibitor, Mdivi-1, affected the protective function of AS-IV on mitochondrial quality and mitophagy

**Fig. 4:**
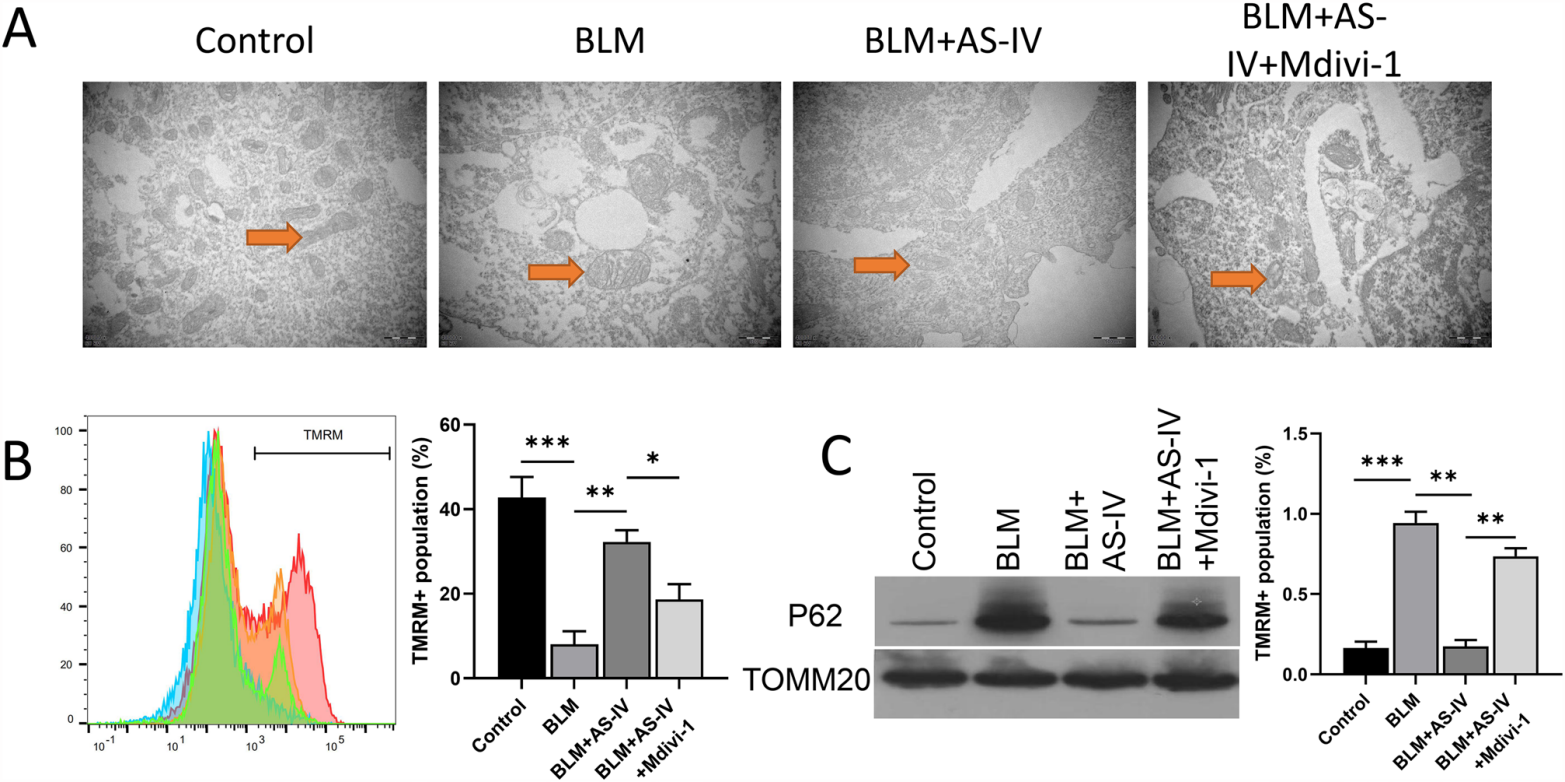
Mitophagy mediators challenged the roles of AS-IV on mitochondrial membrane potential loss and autophagy in BLM-induced VSMCs. AS-IV and Mdivi-1 were applied to treat BLM-induced VSMCs, respectively. **A** The mitochondrial structure was monitored through TEM. **B** TMRM + fluorescence was confirmed with flow cytometer in each group. **C** Western blot was used to assess expression changes of P62 in BLM-stimulated VSMCs. *P < 0.05, **P < 0.01, ***P < 0.001

Likewise, through rescue experiments, we further verified whether autophagy/mitophagy mediators can change the impacts of AS-IV on mitochondrial morphology, mitochondrial membrane potential, and autophagy in BLM-induced VSMCs. As shown in Fig. 4A, mitophagy was inhibited by Mdivi-1. The mitochondria were swollen and their cristae were destroyed (Fig. A). Flow cytometry results showed that Mdivi-1 treatment hindered the protective effect of AS-IV on mitochondrial membrane potential loss (Fig. 4B). The results of Western blot showed that expression of P62 induced by BLM was significantly up-regulated, and the expression level of P62 was significantly down-regulated after AS-IV treatment. (Fig. 4C). In summary, these findings revealed that the mitophagy mediator can regulate AS-IV’s role on mitochondrial quality in BLM-induced VSMCs.

### Parkin-dependent mitophagy attenuated the cellular senescence induced by BLM

**Fig. 5:**
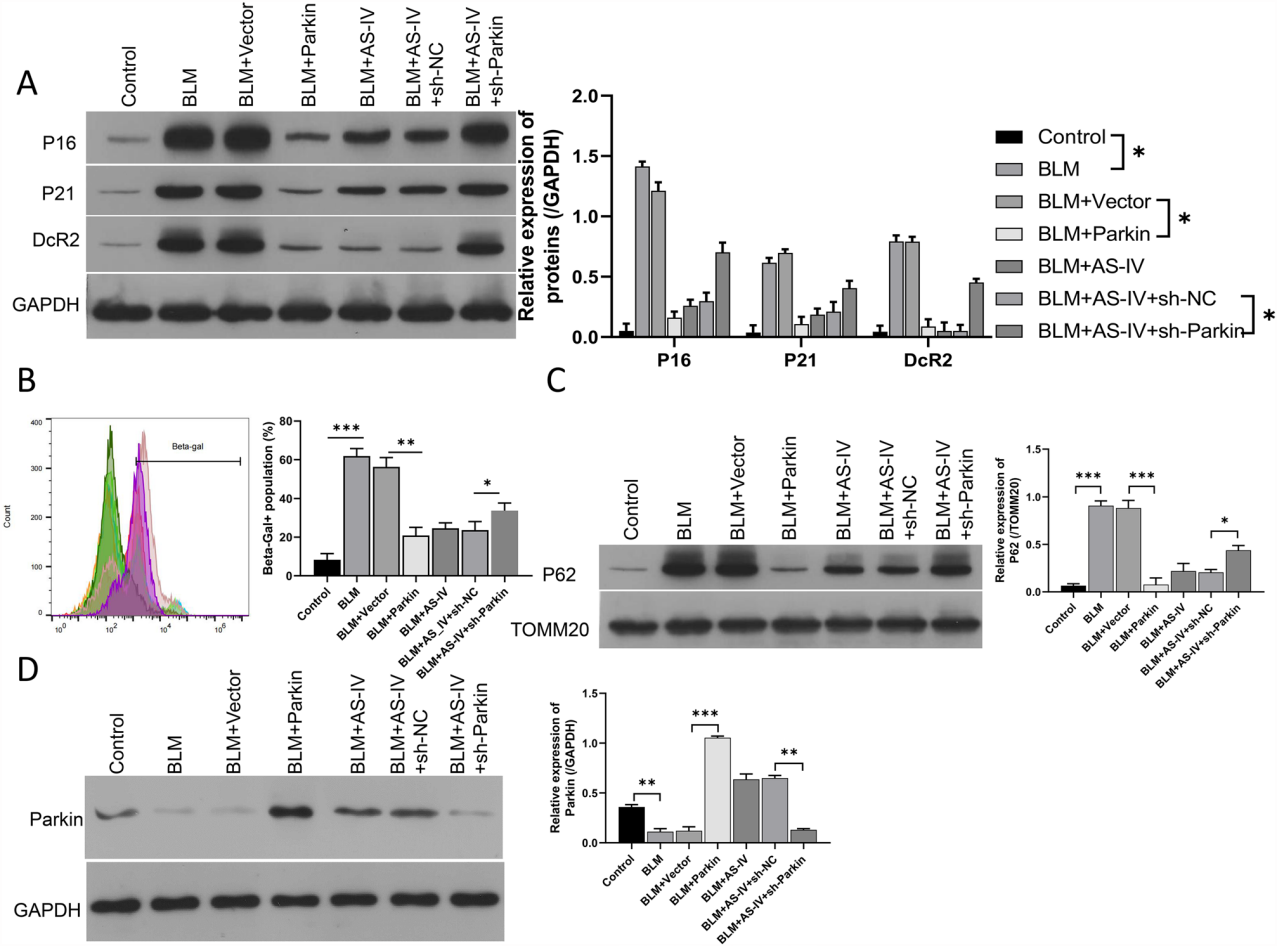
Parkin-dependent mitophagy attenuated the cellular senescence induced by BLM. **A** Expression changes in p16, p21, DcR2 were monitored by Western blot. **B** The change in TMRM + fluorescence was confirmed through flow cytometer. **C** Western blot was used to assess the changes of p62 expression. **D** Western blot was used to evaluate expression of Parkin in VSMCs. *P < 0.05, **P < 0.01, ***P < 0.001

As concluded above, mitophagy acts as a mediator in cellular senescence induced by BLM. Therefore, we further explored whether overexpression or knockdown of Parkin could affect the effects of BLM-induced VSMC senescence. First, changing expression of Parkin in VSMCs was done. As shown in Fig. 5A, overexpression of Parkin or AS-IV delivery significantly downregulated p16, p21, and DcR2 in BLM-induced VSMCs, while, knockdown of Parkin reversed the suppression of p16, p21, and DcR2 by AS-IV (Fig. 5A). Flow cytometry data also disclosed that Parkin overexpression or AS-IV dramatically rescued the mitochondrial membrane potential loss in BLM-induced VSMCs (Fig. 5B), while, knockdown of Parkin impaired the inhibitive role of AS-IV on P62 expression (Fig. 5C). As shown in Fig. 5D, Parkin overexpression reversed the suppression by BLM. Figure 5D also presented that the elevation of Parkin expression induced by AS-IV treatment in VSMCs was reversed by Parkin shRNA (Fig. 5D). As a whole, we showed that AS-IV repressed senescence and activated autophagy in BLM-induced VSMCs by upregulating Parkin.

### AS-IV attenuated senescence and mitophagy in the aging mouse model

**Fig. 6:**
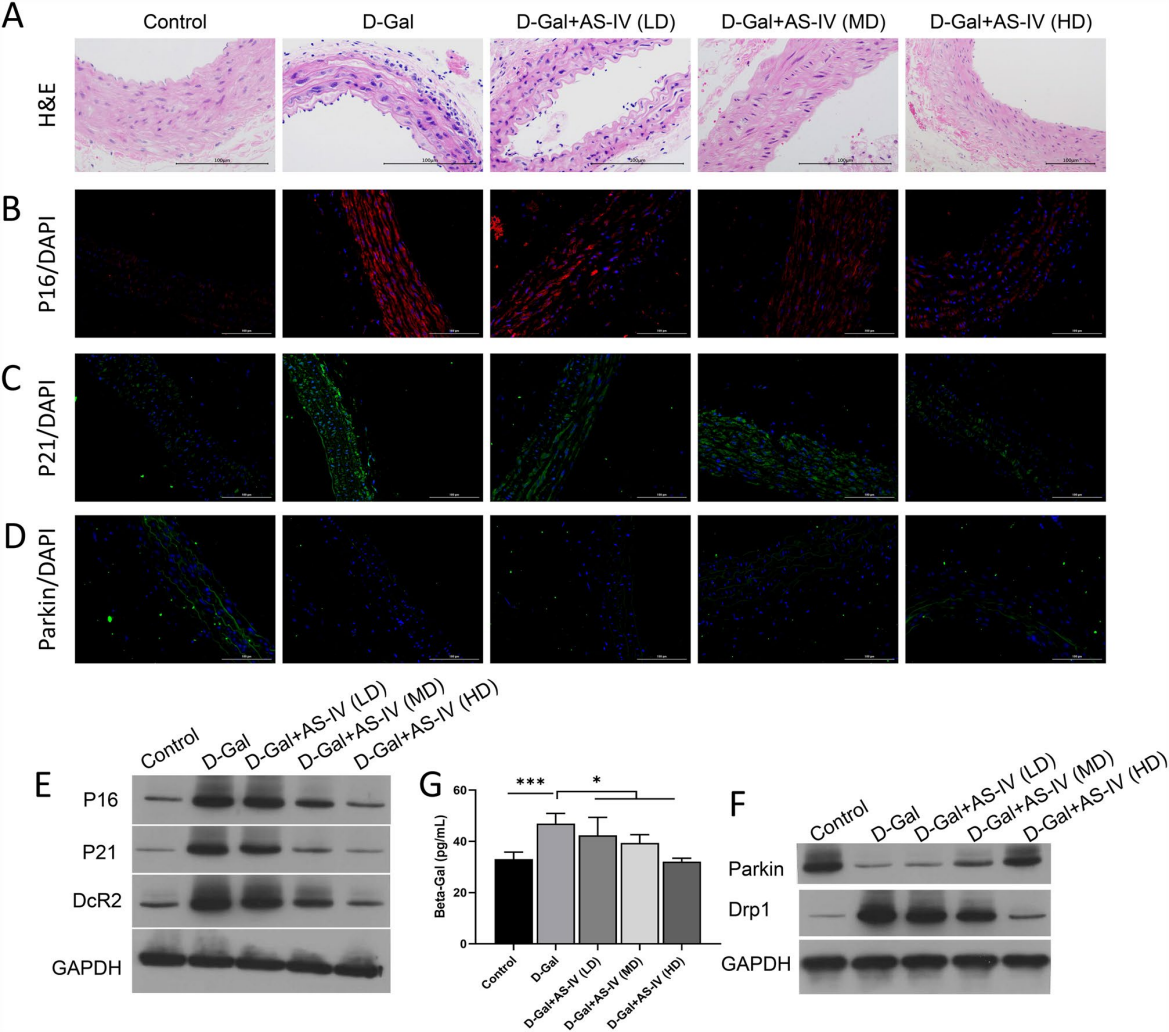
AS-IV attenuated senescence and mitophagy in the aging mouse model The aging mouse model was induced by d-Gal, and then given three doses of AS-IV. **A** Pathological change of vascular tissue was evaluated through H&E staining. Magnification, 200 × , scale bar = 100 μm. **B**–**E** Expressions of p16 (**B**), p21 (**C**), and Parkin (**D**) were accessed by immunofluorescence and Western blot (E). Magnification, 200 × , scale bar = 100 μm. **E** Western blot analysis shows the changes in p16, p21, DcR2 expressions. **G** The concentration of β-galactosidase was determined through the ELISA kit. **F** Western blot was also used to confirm p62 and Drp1 expressions. *P < 0.05, ***P < 0.001

More importantly, we also investigated the influence of AS-IV on the senescence and mitophagy in the aging mouse model. H&E staining results showed that the vascular tissue of the model group (D-Gal) showed a large amount of inflammatory cell infiltration, nuclear staining deepened, and the nucleus showed signs of enlargement. This phenomenon gradually changed with the treatment of AS-IV (Fig. 6A). The results of immunofluorescence and Western blot showed that expressions of P21, p16, and DcR2 were up-regulated in the d-Gal group, while expressions of P21, p16, and DcR2 were inhibited by AS-IV treatment (Fig. 6B–E). Further kit test results showed that the level of β-galactosidase significantly decreased with the increase of the AS-IV dose (Fig. 6G). Western blot results showed that the expression level of Parkin was decreased in the d-Gal group, while AS-IV treatment led to the reverse of Parkin expression, while Drp1 showed the opposite trend (Fig. 6F). The results suggest that there is a correlation between Parkin-mediated mitophagy and d-Gal-induced senescence.

## Discussion

CVDs refers to ischemic or hemorrhagic diseases of the heart, brain and systemic tissues caused by hyperlipidemia, blood viscosity, atherosclerosis, and hypertension [23]. CVDs are characterized by high morbidity, high recurrence, high mortality, and high disability rate[24]. As a multi-factor disease, the pathogenesis of CVDs is increasingly complicated. Therefore, further research on the pathogenesis and effective drugs of CVDs is of great significance for its treatment.

Cellular senescence is the phenomenon in which cells stop dividing due to irreversible cell cycle arrest [1]. It has been reported that VSMC senescence is a key mechanism in the pathogenesis of CVDs [25]. VSMC is the main cell type in vascular walls, and the functional status and activity of VSMCs can directly affect the structure and function of the vascular wall. Phenotypic transformation and functional changes of VSMCs with aging can result in pathological changes of vascular senescence, such as thickening of the vascular wall, decreased elasticity, and enlargement of the lumen [8]. BLM is a chemotherapeutic agent that has been reported to induce cell senescence [26]. To investigate the changes in senescent-related characteristics of VSMCs and its possible regulatory factors, we used BLM to construct a senescent cell model. The main features of cell senescence include increased activity of SA-β-gal, formation of a senescent-associated heterochromatin focus (SAHF) in the nucleus, increased expression of DCR2, and activation of p16 and p21 pathways [27]. Our results revealed that the introduction of BLM can cause an increase of these senescent -related indicators in VSMCs, indicating that BLM can induce VSMC senescence. In our current research, we also used BLM to induce VSMC senescence.

Studies revealed that AM has a wide range of biological activities and curative effects, and has a broad prospect in the treatment of respiratory, digestive, cardiovascular, and immune system diseases [28]. The active substances of AM have been confirmed to play positive roles in the regulation of the oxidative/antioxidant balance and inflammatory response [19]. Among them, AS-IV, as the primary active ingredient of AM, has antiviral, metabolic regulation, antioxidant, tumor inhibition, and other biological functions [29, 30]. With the deepening of AS-IV researches, AS-IV has vital pharmacological effects in the remedy of CVDs, anti-hyperglycemia, anti-aging, and other aspects [31, 32]. Thus, AS-IV has a certain anti-aging role. In our study, the results showed that AS-IV treatment can downregulate p16, p21, and DcR2 and weaken SA-β-galactosidase and SAHF in BLM-induced VSMCs, suggesting that AS-IV can alleviate VSMC senescence. In vivo results also revealed that AS-IV can delay senescence and improve mitochondrial injury in the aging mouse model.

There are multiple causes of cell senescence, such as DNA damage, ROS production caused by oxidative stress, and activation of proto-oncogenes [33]. The study proved that AS-IV can effectively reduce endogenous ROS production [34]. The production of intracellular ROS mainly comes from the mitochondrial respiratory chain. It was reported that mitochondrial dysfunction is both a sign of aging and an initial cause of aging [35]. Mitochondria are found in most cell types and are the main productive structures. Mitochondrial fusion and division are the basis of mitochondrial function [36]. Under normal conditions, mitochondrial fusion and division maintain a dynamic balance to maintain the stable morphology, structure, and function of mitochondria in cells [37]. Currently, mitochondrial fusion proteins include MfN1 and Mfn2, and mitochondrial division proteins include DRP1 [38]. In our study, we discovered that AS-IV can downregulate Drp1 and upregulate Mfn2 in BLM-induced VSMCs, indicating that AS-IV can maintain the balance of mitochondrial fusion and division in the process of VSMC senescence. Moreover, our results verified that the mitochondrial division inhibitor (Mdivi-1) can reverse the inhibition of AS-IV on BLM-induced VSMC senescence, indicating the importance of mitochondrial fusion and division in the prevention of AS-IV-mediated BLM-induced VSMC senescence.

Autophagy is a self-protection mechanism by which eukaryotic cells can remove cellular cohesion and damaged organelles to maintain homeostasis [39]. With the deepening of autophagy-related studies, autophagy has become the key regulatory mechanism of cellular senescence [6]. In mammals, autophagic protein deficiency can cause the accumulation of misfolded proteins and aberrant mitochondria in cells, leading to premature senescence and organ dysfunction [40]. Autophagy and senescence are mediated by multiple signaling pathways. Among them, mTOR, a serine-threonine protein kinase, is the key to the regulation of cell senescence and autophagy [41]. As a crucial negative regulator of autophagy, mTOR can induce autophagy by regulating the production of Atg1/ULK [42]. Study revealed that mitophagy can participate in the pathophysiological processes of various CVDs [43]. In our study, we further proved that AS-IV can downregulate mitochondrial P62 and upregulate mitochondrial LC3 in BLM-induced VSMCs, suggesting that AS-IV can enhance autophagy in the process of VSMC senescence.

Furthermore, we also screened AS-IV-mediated mitophagy-related proteins in BLM-induced VSMCs and found that Parkin has the greatest regulating potential in AS-IV-mediated senescence VSMC delay. Parkin, as an E3 ubiquitin ligase, can degrade misfolded proteins by the ubiquitin proteasome pathway [44]. Also, Parkin, as a potential anti-aging factor, can prevent inflammation and fibrosis and alleviate diabetes-associated myocardial and nerve injury [45, 46]. Research also uncovered that Parkin is related to VSMC senescence [47]. Our current study further disclosed that AS-IV can repress senescence and mitochondrial injury and active autophagy in BLM-induced VSMCs by upregulating Parkin.

## Conclusions

**Fig. 7:**
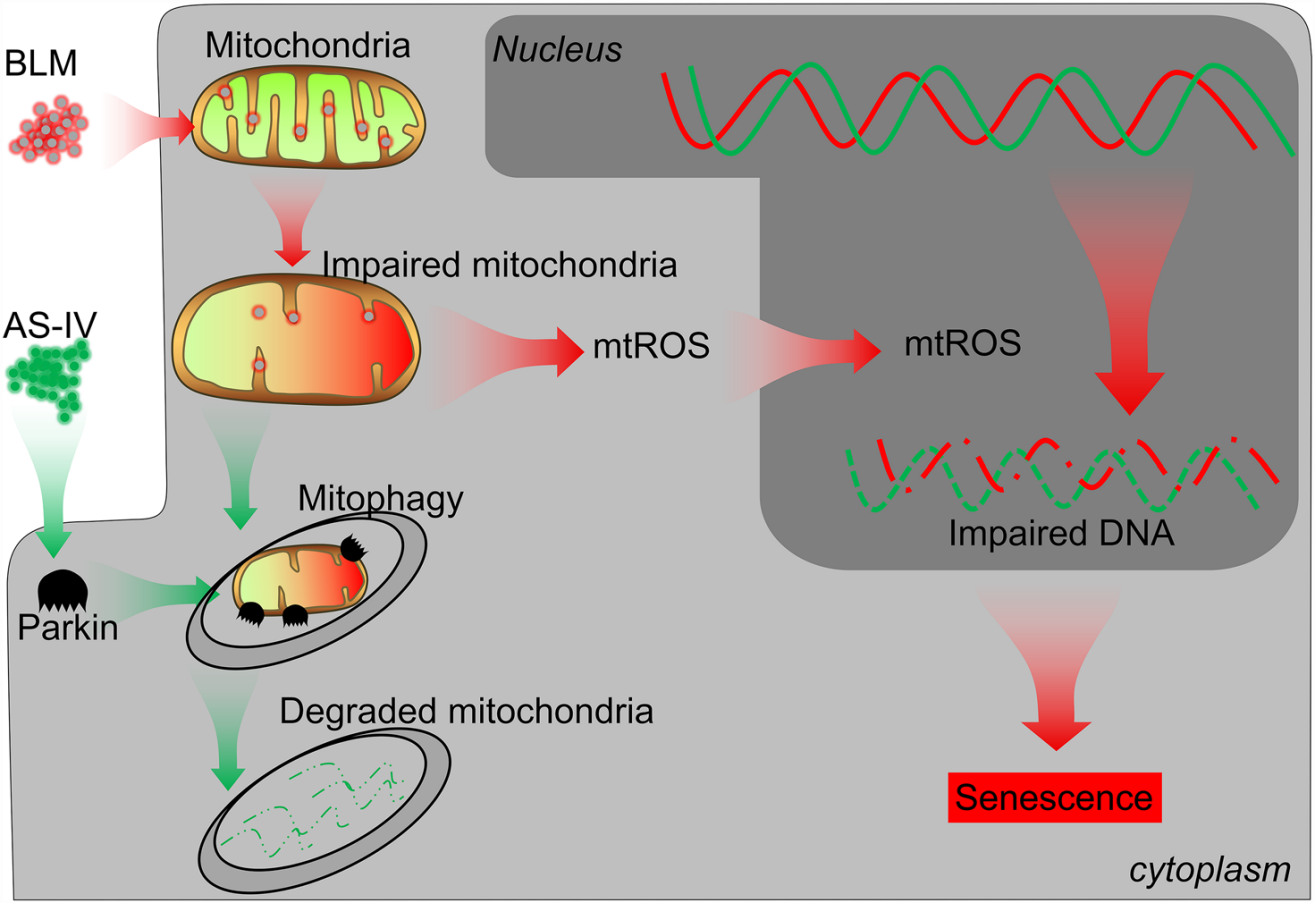
Graphical summary

Our current study demonstrated that AS-IV prevented cellular senescence through mediating mitophagy in VSMCs and vascular tissues via regulating Parkin expression (Fig. 7). This study suggested that AS-IV-mediated Parkin might be a latent therapeutic agent and target for VSMC senescence.

## Supplementary Information

Below is the link to the electronic supplementary material.

Figure S1. The knockdown and overexpression of Parkin in VSMCs. Expression was significantly altered after transfected with Parkin overexpression plaism and shRNA
