# Supplementary figures and images for: Astragaloside IV alleviates senescence of vascular smooth muscle cells through activating Parkin-mediated mitophagy
Source: Hum Cell. 2022 Aug 4;35(6):1684–96. doi: 10.1007/s13577-022-00758-6 (PMC9515037; doi:10.1007/s13577-022-00758-6)

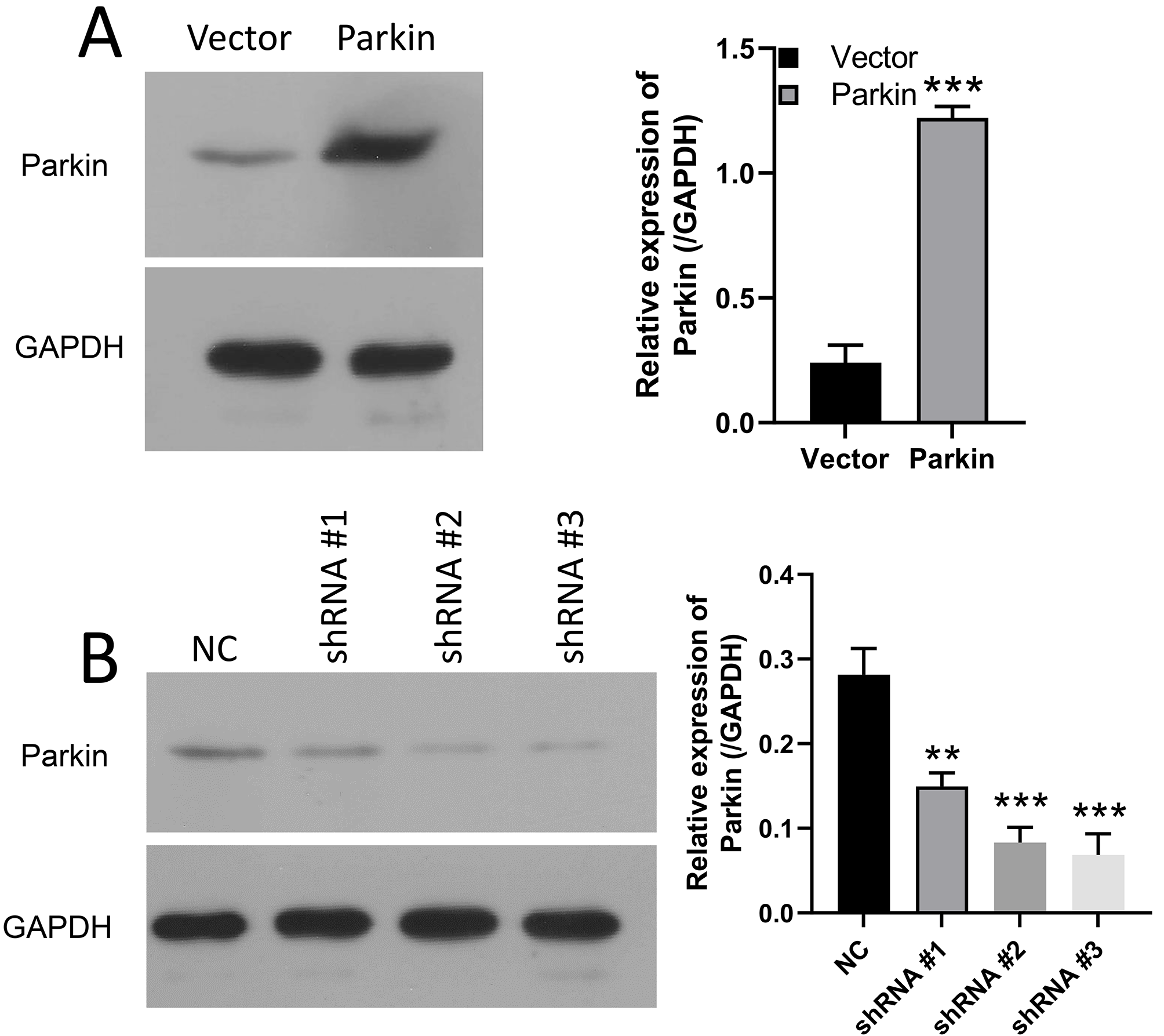

Supplement: Supplementary file 1 — Figure S1. The knockdown and overexpression of Parkin in VSMCs. Expression was significantly altered after transfected with Parkin overexpression plaism and shRNA [file 13577_2022_758_MOESM1_ESM.tif]
